# Changes in compassion and fears of compassion during the COVID-19 pandemic: Findings of a multinational study

**DOI:** 10.1192/j.eurpsy.2023.320

**Published:** 2023-07-19

**Authors:** M. Matos, C. Compassion & Covid-19

**Affiliations:** 1 Center for Research in Neuropsychology and Cognitive and Behavioral Intervention (CINEICC), University of Coimbra, Coimbra, Portugal; 2 Multinational, Multinational, Multinational

## Abstract

**Introduction:**

Cross-sectional data has shown that compassion for self and from others may be a protective factor for greater psychosocial wellbeing in the COVID-19 pandemic whilst fears of compassion act as a risk factor for experiencing mental health difficulties.

**Objectives:**

The current study sought out to explore the natural fluctuation of compassion (for self, for others and from others) and of fears of compassion (for self, for others and from others) across time during the COVID-19 pandemic in a multinational community sample.

**Methods:**

Data from 4057 participants from 21 countries was collected at 3 time points during the pandemic (baseline, 3 months and 6 months). Other than demographic variables, participants completed the Compassionate Engagement and Action Scales and the Fears of Compassion Scales. Multilevel latent growth modelling was used to investigate the main aims.

**Results:**

There was a significant increase in compassion for self and from others, whilst compassion for others remained unchanged throughout the 3 time points [Chi square 349.30(df=50) p< .001; RMSEA .035; CFI .93; TLI .91; SRMR (within) .043; SRMR (between) .70]. Fears of self-compassion and compassion for others significantly reduced throughout the pandemic whilst fears of compassion from others remained stable [Chi square 406.57(df=50) p< .001; RMSEA .038; CFI .96; TLI .94; SRMR (within) .042; SRMR (between) .35].

**Conclusions:**

The findings from this study seem to suggest that in a period of shared suffering people from multiple countries and nationalities tend to become more compassionate and less afraid of and resistant to compassion for and from others.

**Disclosure of Interest:**

None Declared

